# Supplementary material for: mRNA and miRNA Expression Analysis Reveal the Regulation for Flower Spot Patterning in Phalaenopsis ‘Panda’
Source: Int J Mol Sci. 2019 Aug 30;20(17):4250. doi: 10.3390/ijms20174250 (PMC6747512; doi:10.3390/ijms20174250)
Supplement: Supplementary file 1 [file ijms-20-04250-s001.pdf]

Supplementary Materials:

Table S1. Summary of transcriptome sequencing data.

Sample	Raw Reads	Clean Reads	Clean Base (Gb)	Q20 (%)	Q30 (%)	Clean Reads Ratio (%)
Nonspot-A	55,765,564	44,632,028	6.69	98.03	93.91	80.04
Nonspot-B	57,355,802	45,110,536	6.77	97.93	93.61	78.65
spot-A	57,715,006	45,380,086	6.81	98.03	93.91	78.63
spot-B	57,355,434	44,434,116	6.67	97.91	93.53	77.47

Q20 (%): the ratio of bases with Phred values greater than 20 to the total base. Q30 (%): the ratio of bases with Phred values greater than 30 to the total base.

Table S2. Summary of mapping ratio.

Sample	Total Mapping Ratio	Uniquely Mapping Ratio
Nonspot-A	51.71%	29.83%
Nonspot-B	55.81%	32.16%
spot-A	58.38%	33.90%
spot-B	55.98%	32.18%

Table S3. Functional Annotation in NR, KEGG, GO database.

Total	NR	GO	KRGG
22,188	17,871	15,588	19,864

Table S4. The KEGG analysis of DEGs and the most enrichment pathway (top 20).

NO.	Pathway	Gene_Number	Backgroud_Gene_Number	RichFactor	Q-value
1	Photosynthesis - antenna proteins	12	18	0.6666667	2.62×10^{-13}
2	Phenylpropanoid biosynthesis	45	512	0.0878906	2.94×10^{-10}
3	Metabolic pathways	191	4806	0.039742	1.37×10^{-7}
4	Biosynthesis of secondary metabolites	109	2430	0.044856	2.31×10^{-6}
5	Photosynthesis	16	116	0.137931	2.31×10^{-6}
**6**	**Flavonoid biosynthesis**	**12**	**105**	**0.114286**	**5.28×10^{-4}**
7	Glycerolipid metabolism	19	266	0.0714286	2.14×10^{-3}
8	Porphyrin and chlorophyll metabolism	11	123	0.0894309	7.44×10^{-3}
9	Starch and sucrose metabolism	34	701	0.0485021	1.11×10^{-2}
10	ABC transporters	15	235	0.0638298	2.23×10^{-2}
11	Plant-pathogen interaction	36	797	0.0451694	2.23×10^{-2}
12	Amino sugar and nucleotide sugar metabolism	19	341	0.0557185	2.48×10^{-2}
13	Regulation of autophagy	10	156	0.0641026	8.79×10^{-2}
14	Terpenoid backbone biosynthesis	9	137	0.0656934	9.90×10^{-2}
15	Stilbenoid, diarylheptanoid and gingerol biosynthesis	7	100	0.07	1.40×10^{-1}
**16**	**Phenylalanine metabolism**	**8**	**132**	**0.060606**	**1.90×10^{-1}**
17	Cyanoamino acid metabolism	9	159	0.0566038	1.93×10^{-1}
18	Tryptophan metabolism	5	66	0.0757576	2.02×10^{-1}
19	Limonene and pinene degradation	5	73	0.0684932	2.68×10^{-1}
20	Glycerophospholipid metabolism	10	202	0.049505	2.68×10^{-1}

Table S5. Summary of miRNA transcriptome sequencing data.

Sample	Raw Tag	Clean Tag	Clean Base Ratio (%)
Nonspot-A	26,663,561	23,352,936	87.58
Nonspot-B	26,865,118	24,896,769	92.67
spot-A	28,739,122	26,605,438	92.58
spot-B	27,644,225	25,926,370	93.79

Table S6. Summary of mapping ratio of clean tag.

Sample	Clean Tag	Total Mapped Tag	Percentage (%)
Nonspot-A	23,352,936	18,062,942	77.35

Nonspot-B	24,896,769	19,039,072	76.47
spot-A	26,605,438	21,395,134	80.42
spot-B	25,926,370	19,516,924	75.28

Table S7. mRNA QPCR primer.

Primers	Primer Sequences (5'–3')
PeAct4_real_F	TTGTGAGCAACTGGGATGACAT
PeAct4_real_R	GCCACGCGAAGTTCATTGT
PeMYB2_real_F	ATGGTTGTTACCTTCCTGATGATGAGAAC
PeMYB2_real_R	TTCCCACCAGTCCTCTCCTCCTTC
PeMYB7_real_F	CAAGGGAGCATGGACCAAGGAAG
PeMYB7_real_R	TTTGAGATCGGGACGGAGGTAAT
PeMYB12_real_F	AACAAAAGTAGACGCAGCACTACCAAC
PeMYB12_real_R	TTGGGACGGATCATTTCAGCC
PeMYB16_real_F	ACTTTCCCTCAGCAGCATCTACC
PeMYB16_real_R	GGCTTCCAGCTTGGCTATTATCT
PeCHI_real_R	GGTGATAAAGGAGATCAAGGGTT
PeCHI_real_R	GCTGTAGAAGAAGTTGCAGGAAA
PeF3H_real_F	TCATTACCTGAGCAACGGTCGG
PeF3H_real_R	AACGCCGCGATCGAAAGCC
PeDFR_real_F	TTGAGGATATGTTTGATGATGCTATTAAG
PeDFR_real_R	TTCCTCAGTGTTGGGCGGTAAGAG
PeANS_real_F	TGCCACAGCGTTCTTCACCG
PeANS_real_R	AAACCGCCCACGAGATCCGTAC
PeC4H_real_F	TGCTGCCATTGAGACAACCCTGTG
PeC4H_real_R	GCGATGGATGTCCGGCTCTGTGA
PeFLS_real_F	GAGTACGGGAGGCAGATGGTGAG
PeFLS_real_R	CGGGTTGCGGGCATTTAGGATAG
PeF3'H_real_F	GCCCTGCTTGGCTTGGCTTGATT
PeF3'H_real_R	TCCACCATTGACTTCGCCGTTCT
Pe4CL_real_F	GGGCCAGTGCTATCAATGTGCTT
Pe4CL_real_R	CCAGGCTGATTGTAGCCAAGGGA
Pe4CL2_real_F	GGGTACGTGGATGATGACGAGGAG
Pe4CL2_real_R	GAGACTACGGCAGAATCGGCTATGTT
PebHLH_real_F	TCAACTCCACAATGGCTAATGGCAAG
PebHLH_real_R	ATTTTATTTCACGAATGCTGGCTCTC

Table S8. miRNA QPCR primer.

Primers	Primer Sequences (5′–3′)
PeU6_F	CATCCGATAAAATTGGAACGA
PeU6_R	TTTGTGCGTGTCATCCTTGCG
PemiR858_F	GCGGGCTTTCGTTGTCTGTT
PemiR858_R	GTGCAGGGTCCGAGGT
PemiR858-RT	GTTGGCTCTGGTGCAGGGTCCGAGGTATTCGCACCAGAGCCAACAAGGTC
PemiR156g_F	GCGGGCGCTCTCTAGACTTCT
PemiR156g_R	GCTCTCTAGACTTCTGTCATC
PemiR156g-RT	GTTGGCTCTGGTGCAGGGTCCGAGGTATTCGCACCAGAGCCAACGATGAC
